# No evidence of hepatitis E virus-associated central nervous system infections in a Vietnamese multicenter cohort

**DOI:** 10.1016/j.ijregi.2025.100793

**Published:** 2025-10-22

**Authors:** Do Van Dong, Vu Viet Sang, Nghiem Xuan Hoan, Nguyen Thi Khanh Linh, Hoang Xuan Quang, Tran Thi Lien, Van Dinh Trang, Le Huu Song, Thirumalaisamy P. Velavan

**Affiliations:** 1 Institute of Tropical Medicine, Universitätsklinikum Tübingen, and German Centre for Infection Research (DZIF), Tübingen, Germany; 2 108 Military Central Hospital, Hanoi, Vietnam; 3 Vietnamese German Centre for Medical Research (VG-CARE), Hanoi, Vietnam; 4 103 Military Hospital, Hanoi, Vietnam; 5 Viet Tiep Friendship Hospital, Haiphong, Vietnam; 6 Hai Phong University of Medicine and Pharmacy, Haiphong, Vietnam; 7 National Hospital for Tropical Diseases, Hanoi, Vietnam; 8 Faculty of Medicine, Duy Tan University, Danang, Vietnam

**Keywords:** Central nervous system infections, Hepatitis E virus, Neurological manifestations, Cerebrospinal fluid, Molecular diagnostics

## Abstract

**Objectives:**

Hepatitis E virus (HEV), traditionally associated with acute hepatitis, has been increasingly recognized as a potential cause of neurological disease, particularly in Europe and parts of Asia. However, its role in central nervous system (CNS) infections in Vietnam has not been investigated.

**Methods:**

We retrospectively analyzed cerebrospinal fluid (CSF) samples from 330 patients hospitalized with suspected CNS infections at four tertiary hospitals in Northern Vietnam. Extensive diagnostics, including CSF culture, multiplex polymerase chain reaction (PCR), customized PCR assays, and 16S nanopore sequencing, identified a pathogen in 27% of cases. All samples were additionally screened for HEV RNA using nested PCR targeting the ORF1 region.

**Results:**

No HEV RNA was detected in this study. In our previous investigations, the most common pathogens identified were *Mycobacterium tuberculosis, Klebsiella pneumoniae, Streptococcus suis, Streptococcus pneumoniae, Neisseria meningitidis, Acinetobacter baumannii,* herpes simplex virus type 1, and varicella-zoster virus. These findings suggest that HEV is unlikely to play a major role in CNS infections in this setting, although the lack of serological testing and genotyping limits interpretation.

**Conclusions:**

Given evidence of HEV genotype 3 circulation in Vietnam, future prospective studies combining molecular and serological approaches are warranted to clarify the potential contribution of HEV to neurological disease.

## Highlights

• Multicenter cohort screened for hepatitis E virus (HEV) in central nervous system infections in Vietnam.
• No HEV RNA detected in 330 cerebrospinal fluid samples.
• Findings suggest a limited role of HEV in neurological infections.
• Future studies should integrate serology and genotyping approaches.

## Introduction

Central nervous system (CNS) infections are a leading cause of hospitalization worldwide, with clinical presentations ranging from meningitis and encephalitis to meningoencephalitis. CNS infection causative pathogens include bacteria, fungi, parasites, and numerous neurotropic viruses. In Vietnam, viruses accounted for 14% of identified CNS infections, with Japanese encephalitis virus, herpes simplex virus, and enterovirus being the most common [1]. Yet, in nearly two-thirds of cases, no causative pathogen was identified. These findings highlight the diagnostic challenges and suggest that some “unknown cause” cases may be attributable to under-recognized viral pathogens. Hepatitis E virus (HEV), classically associated with hepatitis, has increasingly been reported as a cause of neurological disease. Case reports primarily from Europe and Asia describe HEV-associated meningoencephalitis, neuralgic amyotrophy, Guillain-Barré syndrome, and encephalitis [2]. The global distribution of HEV genotypes varies: genotypes 1 and 2, transmitted via the fecal–oral route, predominate in low- and middle-income countries and are typically linked to large waterborne outbreaks [3] whereas genotypes 3 and 4, more common in high-income countries, are associated with zoonotic transmission, chronic infections, and extrahepatic manifestations, including neurological disease [4]. While HEV circulates in Vietnam [5,6], its role in CNS infections has not been studied. We therefore screened cerebrospinal fluid (CSF) samples from a large multicenter cohort of patients hospitalized with CNS infections in Northern Vietnam to investigate whether HEV contributes to the local burden of neurological disease.

## Methods

This retrospective study included 330 patients hospitalized with suspected CNS infections at four referral hospitals in Hanoi, Northern Vietnam: 108 Military Central Hospital (108 MCH), National Hospital for Tropical Diseases (NHTD), 103 Military Hospital (103 MH), and Viet Tiep Friendship Hospital (VT). Written informed consent was obtained from patients or their guardians. The study protocol was approved by the institutional review board of 108 MCH (Protocol No. 108MCH/RES/MENTNGITIS-V-D3-25042017) and conducted in accordance with international Good Clinical Practice (GCP) guidelines. Cerebrospinal fluid (CSF) samples (n = 330) were analyzed using a combination of standard diagnostics (CSF culture, in-house polymerase chain reaction [PCR]), the BioFire FilmArray Meningitis/Encephalitis panel, customized PCR assays, and 16S rRNA sequencing with Oxford Nanopore Technology, as previously described [7,8]. For HEV screening, RNA was extracted from CSF and tested using a nested PCR targeting a conserved region of HEV ORF1, as described earlier [5].

## Results

A total of 330 patients were included: 52 from 108 MCH, 136 from NHTD, 40 from 103 MH, and 102 from VT. Patient characteristics are summarized in Table 1. Routine and advanced diagnostics identified a pathogen in 90 of 330 cases (27%), as reported in our previously published studies [[7], [8], [9]]. Clinical features such as fever, neck stiffness, vomiting, and altered mental status were more common in patients with confirmed infections than in those with unknown etiologies (Table 1). CSF cell counts, protein, and glucose levels also correlated with pathogen-positive cases (Table 1). Importantly, no HEV RNA was detected in any CSF samples, regardless of whether another pathogen was identified. Because this was a retrospective analysis, paired serum samples were not available, as only cerebrospinal fluid was stored during the initial diagnostic work-up. Therefore, no serological testing for anti-HEV immunoglobulin (Ig)M or IgG could be performed.

**Table 1:** Overview of patients with CNS infections (n = 330).

Patient characteristics		All patients with CNS infection n = 330 (%)	Patients with a causative pathogen identified n = 90 (%)	No causative pathogen identified n = 240 (%)	*P*-value
**Demographics**					
Age (Mean ± SD)		54 ± 19	53 ± 19	55 ± 19	0.458
Male sex - n (%)		225 (68)	67 (74)	158 (66)	0.173
**Underlying conditions – n (%)**					
Hypertension		90 (27)	19 (21)	71 (30)	0.161
Diabetes		62 (19)	5 (6)	57 (24)	**<0.001**
Cardiac disease		19 (6)	2 (2)	17 (7)	0.155
Alcoholism		17 (5)	9 (10)	8 (3)	**0.023**
Chronic liver disease		18 (6)	4 (4)	14 (6)	0.788
Chronic lung disease		17 (5)	7 (8)	10 (4)	0.26
Kidney disease		15 (5)	2 (2)	13 (5)	0.372
Immuno-suppressive drugs		13 (4)	3 (3)	10 (4)	1
Cancer		13 (4)	0 (0)	13 (5)	**0.023**
HIV-positive		9 (3)	3 (3)	8 (3)	1
**Risk factors – n (%)**					
Post neurosurgery		29 (9)	11 (12)	18 (8)	0.258
Head trauma		31 (9)	8 (9)	23 (10)	1
CSF shunt		5 (2)	0 (0)	5 (2)	0.328
**Clinical features - n (%)**					
Fever (>37.5°C)		273 (83)	82 (91)	191 (80)	**0.021**
Headache		221 (67)	66 (73)	155 (65)	0.17
Neck stiffness		207 (63)	70 (78)	137 (57)	**0.001**
Nausea/Vomiting		99 (30)	36 (40)	63 (26)	**0.022**
Seizure		29 (9)	5 (6)	24 (10)	0.293
Focal neurologic deficits		20 (6)	6 (7)	14 (6)	0.981
Glasgow coma score		13 ± 2	13 ± 2	13 ± 2	**0.013**
Altered mental status		142 (43)	56 (62)	86 (36)	**<0.001**
At least 2/4 symptoms recorded (headache, fever, stiff neck, altered mental status)		273 (83)	82 (91)	191 (80)	**0.021**
**Glasgow outcome score n (%)**					
1: Death		32 (10)	8 (9)	24 (10)	0.055
2: Vegetative state		29 (9)	13 (14)	16 (7)	
3: Severe disability		64 (19)	14 (16)	50 (21)	
4: Moderate disability		62 (19)	11 (12)	51 (21)	
5: Mild or no disability		143 (43)	44 (49)	99 (41)	
**Cerebrospinal fluid parameters**					
Cell count (cells/mm^3^)	Mean ± SD	1032 ± 5897	3220 ± 10800	202 ± 1240	**0.01**
Protein (g/l)	Mean ± SD	1.56 ± 2.78	2.66 ± 3.37	1.15 ± 2.41	**<0.001**
Glucose (mmol/l)	Mean ± SD	4.16 ± 2.96	3.05 ± 2.82	4.57 ± 2.91	**<0.001**

CNS, central nervous system.

## Discussion

Despite extensive molecular screening, HEV was not detected in this multicenter cohort of CNS infections in Vietnam. This suggests that HEV is unlikely to be a significant contributor to CNS infections in this setting. Nevertheless, the absence of detections should be interpreted with caution for several reasons. First, RNA positivity in CSF is often transient, and HEV infection may be missed without concurrent serological testing. Serological assays for anti-HEV IgM and IgG are essential complements to RNA detection for diagnosing HEV-associated neurological disease [10]. The lack of paired serum/CSF samples in our study was a major limitation. Second, geographical and epidemiological variation in circulating HEV genotypes may influence the neurological disease burden. HEV genotypes 1 and 2 predominate in low- and middle-income countries, whereas genotypes 3 and 4, more common in high-income countries, are frequently linked to extrahepatic and neurological manifestations. Notably, genotype 3 is also an established zoonotic reservoir in Vietnam, as demonstrated in our previous studies among pregnant women and in patients with chronic Hepatitis B virus (HBV) infection [5,6]. Nevertheless, our negative findings in CNS infections may reflect the local genotype distribution rather than the absence of HEV neurotropism globally. Third, although case reports from Europe and parts of Asia have described HEV-associated meningoencephalitis, Guillain-Barré syndrome, and neuralgic amyotrophy, these remain relatively rare. Negative findings from population-level cohorts such as ours are equally valuable in defining the regional epidemiology of HEV.

In this first multicenter investigation of HEV in CNS infections in Vietnam, no HEV RNA was detected in CSF samples. These findings suggest a limited role for HEV in neurological disease in this setting. However, future prospective studies with paired CSF and serum samples, serological testing, and HEV genotyping are needed to clarify whether HEV contributes to CNS infections in Southeast Asia. Such studies will help determine whether HEV is an under-recognized cause of neurological disease globally and will inform priorities for clinical diagnostics and public health surveillance.

## Declaration of competing interest

All authors have no conflict of interest. The funder has no role in the study design, data collection and analysis, decision to publish or preparation of the manuscript. All authors declared no conflict of interest.
